# Association among Dietary Inflammatory Index, sleep quality and nutritional status in fixed daytime and rotating shift workers

**DOI:** 10.1371/journal.pone.0360075

**Published:** 2026-10-22

**Authors:** Şeyma Sünbül, Perim Fatma Türker

**Affiliations:** 1 Department of Nutrition and Dietetics, Faculty of Health Sciences, Ankara University, Ankara, Türkiye; 2 Kalecik Vocational School, Food Technology Program, Ankara University, Ankara, Türkiye; 3 Department of Nutrition and Dietetics, Faculty of Health Sciences, Acibadem Mehmet Ali Aydinlar University, Istanbul, Türkiye; 4 Department of Nutrition and Dietetics, Graduate School of Health Sciences, Acibadem Mehmet Ali Aydinlar University, Istanbul, Türkiye; University of Guelph, CANADA

## Abstract

Shift work is associated with irregular eating behaviors, increased energy intake, and impaired sleep quality. Poor dietary patterns may elevate systemic inflammation, contributing to long-term metabolic and cardiovascular risks. The aim of this study was to investigate differences in the Dietary Inflammatory Index (DII), sleep quality, dietary intake, and anthropometric and body composition measures between fixed daytime workers and rotating shift workers and to examine the associations among these parameters. This cross-sectional study included 52 male workers employed at a defense industry factory in Ankara, Türkiye, who worked either fixed daytime or rotating shifts. Dietary intake was assessed using 24-h dietary recalls for three non-consecutive days, from which macronutrient and micronutrient intakes were calculated. DII scores were computed to assess the inflammatory potential of the diet. Sleep quality was evaluated using the Pittsburgh Sleep Quality Index (PSQI). Anthropometric measurements, body composition, blood pressure, and biochemical parameters, including fasting plasma glucose, lipid profile (total cholesterol, HDL-C, LDL-C, and triglycerides), C-reactive protein, and homocysteine, were measured using standardised protocols. Rotating shift workers had significantly higher total energy intake and higher daily intakes of fructose, total fat, saturated and monounsaturated fatty acids, cholesterol, and several micronutrients, including vitamin A, folate, iron, calcium, and phosphorus compared with fixed daytime workers (p < 0.05). Mean DII scores did not differ significantly between the groups (Fixed daytime: 0.2 ± 1.86; rotating shift: −0.2 ± 2.25). DII scores were positively correlated with global PSQI score (r = 0.278, p = 0.046), the PSQI sleep duration component score (r = 0.319, p = 0.021), and daytime dysfunction component score (r = 0.287, p = 0.039); however, the association between DII and overall sleep quality was no longer statistically significant after adjustment for potential confounding factors (age, BMI, smoking status, physical activity, and work schedule). These findings suggest that dietary habits, pro-inflammatory dietary patterns, and poor sleep quality may represent co-existing factors associated with metabolic risk, and addressing these factors may contribute to better overall metabolic health.

## Introduction

Shift work is widely used to maintain continuous operations and is commonly organised as fixed or rotating schedules [1–4]. In rotating shift systems, workers alternate between day, evening, and night shifts, which may disrupt the alignment between work–rest patterns and endogenous biological rhythms [5,6]. This circadian misalignment is particularly relevant during evening and night shifts, when individuals remain active during periods normally reserved for rest [6]. Persistent circadian disruption has been associated with alterations in hormonal regulation, blood pressure, and immune function [7], as well as impaired metabolic homeostasis, increased systemic inflammation, and a higher risk of metabolic disorders such as obesity [8].

Nutrition is a key modifiable determinant of systemic inflammation. Dietary patterns characterised by high intakes of whole grains, dietary fiber, fruits, vegetables, polyunsaturated fatty acids, and antioxidant vitamins have been associated with reduced inflammatory responses and protective health effects [9–14]. In contrast, dietary patterns rich in saturated fatty acids, refined carbohydrates, red and processed meats, and sugar-sweetened beverages have been linked to elevated inflammatory markers, including C-reactive protein (CRP) and interleukin-6 (IL-6) [15]. Shivappa et al. developed the Dietary Inflammatory Index (DII) to assess the inflammatory potential of diets, which has been used in many epidemiological and clinical studies. Higher DII scores indicate a more pro-inflammatory dietary profile, whereas lower scores indicate a more anti-inflammatory profile [16,17]. Work schedules can substantially influence eating behaviors and dietary patterns. Shift work has been associated with irregular meal timing, increased nighttime snacking, greater consumption of discretionary foods, and lower intake of core food groups compared with daytime work [18]. These dietary characteristics may contribute to a more pro-inflammatory dietary profile and, consequently, higher DII scores. In a study conducted in a U.S. population, DII scores were reported to be significantly higher among shift workers than among daytime workers [19]. Evidence from occupational populations indicates that shift work is associated with a higher likelihood of sleep disturbances, with rotating shift schedules showing an increased risk of sleep disturbances [20]. These disturbances may be partly related to circadian misalignment and disruption of the sleep–wake cycle associated with shift work [21].

Sleep quality is a multidimensional construct that encompasses not only sleep duration but also sleep latency, sleep efficiency, sleep disturbances, use of sleep medication and daytime functioning [22]. The relationship between dietary inflammatory potential and sleep has been examined predominantly in general populations, with systematic reviews reporting associations between higher DII scores and poorer sleep quality and other adverse sleep outcomes [23–25]. Potential mechanisms underlying this relationship include alterations in inflammatory mediators such as interleukin-6 (IL-6), tumor necrosis factor-alpha (TNF-α), and C-reactive protein (CRP), increased oxidative stress, and dysregulation of melatonin secretion and circadian rhythms through interactions between inflammatory pathways and sleep regulation [26–28]. Evidence from shift-working populations is less consistent. In a study of police officers, associations between E-DII scores and sleep outcomes varied according to the sleep measure and long-term shift schedule [29]. These findings indicate that the relationship between dietary inflammatory potential and sleep in the context of shift work may be complex and warrants further investigation in different occupational populations.

Despite growing interest in the relationship between dietary inflammatory potential and sleep quality, studies simultaneously evaluating dietary inflammatory potential, multidimensional sleep quality, dietary intake, nutritional status, and metabolic parameters among fixed daytime and rotating shift workers remain limited. The present study addresses this gap by comprehensively assessing these parameters among fixed daytime and rotating shift workers employed in the defense industry in Türkiye. This approach provides context-specific evidence from an understudied occupational population.

In this context, the primary objective of this study was to compare dietary inflammatory potential between fixed daytime and rotating shift workers, with the DII score defined as the primary outcome. Secondary objectives were to compare dietary intake, sleep quality, anthropometric and body composition measurements, blood pressure, and biochemical parameters, including fasting plasma glucose, lipid profile, CRP, and homocysteine, between the two work schedules and to examine the association between DII and sleep quality. Given the cross-sectional design, the study was intended to examine associations rather than infer causal relationships. We hypothesized that rotating shift workers would have higher DII scores, poorer sleep quality, and less favorable dietary, anthropometric, body composition, blood pressure, and biochemical profiles than fixed daytime workers and that higher DII scores would be associated with poorer sleep quality.

## Methods

### Study design and participants

This cross-sectional study was conducted at a defense industry factory in Ankara, Türkiye. The overall study period extended from July 2022 to October 2023, while data collection was conducted between 28 October 2022 and 24 May 2023. Workers who met the predefined inclusion criteria and voluntarily agreed to participate were included in the study.

An a priori sample size estimation was performed using G*Power software (version 3.1.9.6), with the DII score designated as the primary outcome. A two-tailed independent-samples t-test was specified to estimate the sample size required to detect a difference in DII scores between fixed daytime and rotating shift workers. Based on a large standardised effect size (Cohen’s d = 0.80) according to Cohen’s conventional benchmarks [30,31], a significance level (α) of 0.05, and a statistical power (1 − β) of 80%, the minimum required sample size was determined to be 52 participants (26 participants per group). Eligible workers at the facility were invited to participate in the study. Among those initially contacted, some declined to participate or withdrew before completing the study procedures. Ultimately, 52 participants (26 fixed daytime workers and 26 rotating shift workers) completed all study assessments and were included in the final analysis. Fixed daytime workers consistently worked the daytime (08:00–16:00), whereas rotating shift workers followed a weekly rotation across three eight-hour shifts: day (08:00–16:00), evening (16:00–24:00), and night (00:00–08:00).

As rotating shift work at the study facility was predominantly performed by male workers, only male workers were included in the study. The inclusion criteria were actively employed at the factory, having at least one year of experience in the current work schedule, and providing written informed consent. Individuals with any physician-diagnosed chronic disease (including liver, kidney, cardiovascular disease, cancer, or psychiatric disorders), as well as those with reported food allergies or food intolerances, were excluded from the study.

### Ethical considerations

The study protocol was approved by the Başkent University Non-Interventional Clinical Research Ethics Committee (Project No: KA22/288; Decision No: 22/134; Date: 06 July 2022). All participants provided written informed consent prior to their participation. The study was conducted in accordance with the principles of the Declaration of Helsinki.

### Data collection

Data were collected through face-to-face interviews using standardised questionnaires administered by trained researchers. These questionnaires captured information on sociodemographic characteristics, lifestyle behaviours, dietary intake and sleep quality. Anthropometric measurements, body composition parameters, blood pressure and biochemical data were obtained in accordance with standardised measurement protocols.

### Anthropometric and body composition measurements

Anthropometric measurements and body composition assessments were performed in the early morning after an overnight fast of at least 8 hours. All measurements were obtained by the same trained dietitian following standardised WHO procedures to minimize inter-rater variability. Body weight (kg), body fat percentage (%), lean body mass (kg), and total body water (%) were measured using a Tanita BC-730 bioelectrical impedance analyzer under standardised conditions [32]. Height, waist circumference, hip circumference, mid-upper arm circumference, and neck circumference were measured using standardised anthropometric procedures [33–35]. Body mass index (BMI, kg/m^2^), waist-to-height ratio, and waist-to-hip ratio were subsequently calculated.

### Dietary assessment

Dietary intake data were collected using 24-h dietary recalls on three non-consecutive days [36]. For fixed daytime workers, dietary recalls were obtained on two working days and one non-working day. For rotating shift workers, dietary recalls were obtained on two working days representing two different shift types within the rotating schedule and one non-working day. All dietary recalls were administered face-to-face by a trained dietitian. Participants received standardised instructions and used the Turkish Food Photograph Atlas to estimate portion sizes [37]. Energy and nutrient intakes were calculated from the dietary recall data using the Nutrition Information System (BeBiS), a dietary analysis software program based on the Turkish food composition database.

### Dietary Inflammatory Index

The Dietary Inflammatory Index (DII) is a measure of the inflammatory potential of the diet, validated according to the effects of dietary components on inflammatory markers [16,17]. In the present study, the conventional DII was calculated using 33 food parameters, including energy. Previous validation studies have demonstrated virtually no degradation in the predictive ability of the DII when calculated using 28 rather than the full set of available food parameters [38]. The components included in the DII calculation were energy, protein, total fat, saturated fat, monounsaturated fat, polyunsaturated fat, omega-3 fatty acids, omega-6 fatty acids, cholesterol, carbohydrate, fiber, caffeine, alcohol, vitamin A, beta-carotene, vitamins B6, B12, C, D, and E, thiamine, riboflavin, niacin, folic acid, iron, magnesium, zinc, selenium, as well as green/black tea, onion, garlic, pepper, and turmeric.

In the present study, the DII scores were calculated using the method described by Shivappa et al. [17]. For the calculation, we subtracted the global average daily consumption amounts from the participants’ average daily intake of foods and nutrients, and divided the result by the standard deviation. We converted these to percentiles to reduce the right skewness of the obtained z-scores. Then, we multiplied the percentile values by 2 and subtracted 1 from the result. We multiplied the centred percentile values obtained after this procedure by the customised Inflammatory Effect Score value of that food and nutrient. This way, we calculated the DII value of each food and nutrient. As a result of the sum of these values, the total DII scores from the foods in the diet of the individuals were calculated [17]. Negative DII values indicate an anti-inflammatory dietary profile, whereas positive values indicate a pro-inflammatory dietary profile.

### Biochemical parameters and blood pressure

Venous blood samples were collected by the workplace nurse in the morning after an overnight fast. Blood samples were analysed at an accredited laboratory using standard automated methods. Laboratory measurements included fasting plasma glucose, total cholesterol, high-density lipoprotein cholesterol (HDL-C), low-density lipoprotein cholesterol (LDL-C), triglycerides, C-reactive protein (CRP), and homocysteine concentrations. Systolic blood pressure (SBP) and diastolic blood pressure (DBP) were measured after a 10-minute rest in the seated position using a calibrated sphygmomanometer. Two consecutive blood pressure measurements were obtained, and the average of these measurements was used for analysis, in accordance with standard clinical guidelines.

### Sleep quality assessment

Sleep quality was assessed using the Pittsburgh Sleep Quality Index (PSQI), a widely used instrument developed by Buysse et al. in 1989 to evaluate sleep quality over the preceding one-month period [23]. The Turkish validity and reliability of the PSQI were established by Ağargün et al. in 1996 [39]. In the present study, the validated Turkish version of the PSQI was administered. The PSQI consists of 19 self-rated items that generate seven component scores: subjective sleep quality, sleep latency, sleep duration, habitual sleep efficiency, sleep disturbances, use of sleep medication, and daytime dysfunction. Each component is scored on a scale ranging from 0 to 3, and the global PSQI score is calculated as the sum of all component scores, yielding a total score between 0 and 21, with higher scores indicating poorer sleep quality [40]. A global PSQI score greater than 5 has been shown to distinguish poor sleepers from good sleepers with 89.6% sensitivity, 86.5% specificity, and substantial diagnostic agreement (κ = 0.75, p < 0.001) [23].

### Statistical analysis

The data were analysed using the Statistical Package for the Social Sciences (SPSS) 25.0 (IBM Corp., NY, USA). Normally distributed continuous variables were summarised as mean ± standard deviation (SD), whereas non-normally distributed continuous variables were summarised as median (interquartile range [IQR]). Categorical variables were presented as frequency (percentage). Normality was assessed using skewness and kurtosis values; values between −1 and +1 were considered indicative of an approximately normal distribution. The independent-samples t-test was used for normally distributed continuous variables, whereas the Mann–Whitney U test was used for non-normally distributed variables. The Pearson chi-square test was used to compare categorical variables between groups. Pearson’s correlation was used to assess associations between normally distributed continuous variables, whereas Spearman’s correlation was used for non-normally distributed variables. Multiple linear regression analysis was performed with global PSQI score as the dependent variable and DII score as the main independent variable. Age, BMI, smoking status, physical activity, and work schedule were included as adjustment variables because they were considered potential confounders of the association between DII and sleep quality, based on their plausible relationships with dietary patterns, inflammatory status, and/or sleep characteristics. Work schedule was additionally included to account for the potential influence of shift-related circadian disruption on sleep quality. Regression results were reported as unstandardised regression coefficients (B) with 95% confidence intervals (CIs), along with standardised regression coefficients (β). The assumptions of multiple linear regression were evaluated using residual diagnostic plots, the Durbin–Watson statistic, and variance inflation factors (VIFs). Residual plots were examined to assess normality, linearity, and homoscedasticity, while multicollinearity was assessed using VIF values. Statistical significance was set at p < 0.05.

## Results

The general characteristics of participants according to shift schedule are presented in Table 1. The mean age was 36.0 ± 6.9 years in fixed daytime workers and 35.6 ± 7.1 years in rotating shift workers. Marital status distributions were identical, with 61.5% of each group being married. Educational attainment was also similar between groups; 65.4% of fixed daytime workers and 69.3% of rotating shift workers had a high school education or below. Smoking was reported by 57.6% of fixed daytime workers and 34.6% of rotating shift workers, while alcohol consumption was reported by 34.6% and 15.4%, respectively. No significant differences were observed between the groups in education level, smoking status, alcohol consumption, number of main meals per day, or snack frequency (all p > 0.05).

**Table 1:** General characteristics of all participants.

Participant Characteristics		Fixed Daytime Workers (n = 26)	Rotating Shift Workers (n = 26)	All Workers (n = 52)	p
Age (years), mean±SD		36.0 ± 6.94	35.6 ± 7.07	35.8 ± 6.94	
Marital status, n (%)	Married	16 (61.5)	16 (61.5)	32 (61.5)	
	Single	10 (38.5)	10 (38.5)	20 (38.5)	
Education level, n (%)	High school or below	17 (65.4)	18 (69.3)	35 (67.3)	0.768
	Associate degree or higher	9 (34.6)	8 (30.7)	17 (32.7)	
Smoking status, n (%)	Yes	15 (57.6)	9 (34.6)	24 (46.2)	0.095
	No	11 (42.4)	17 (65.4)	28 (53.8)	
Alcohol consumption, n (%)	Yes	9 (34.6)	4 (15.4)	13 (25)	0.109
	No	17 (65.4)	22 (84.6)	39 (75)	
Main meals/day, n (%)	2	10 (38.5)	11 (42.3)	21 (40.4)	0.777
	3	16 (61.5)	15 (57.7)	31 (59.6)	
Snacks/day, n (%)	≤1	18 (69.2)	17 (65.4)	35 (67.3)	0.768
	≥2	8 (30.8)	9 (34.6)	17 (32.7)	

p < 0.05; p-values are from Pearson’s chi-square test.

Table 2 presents anthropometric measurements and body composition parameters according to work schedule. No significant differences were observed between groups for BMI, waist circumference, hip circumference, mid-upper arm circumference, neck circumference, or waist-to-hip ratio (all p > 0.05). However, the fixed daytime group had a significantly higher total body water percentage (56.0 ± 4.6%) compared with the rotating shift group (53.6 ± 3.7%) (p = 0.040).

**Table 2:** Anthropometric measurements and body composition according to work schedule.

	Fixed Daytime Workers(n = 26)		Rotating Shift Workers(n = 26)			
Variables	Mean±SD	Min-Max	Mean±SD	Min-Max	t	p
**Anthropometric measurements**						
BMI (kg/m^2^)	24.6 ± 3.06	18.6-31.1	26.5 ± 4.21	17.6-35.3	−1.864	0.068
Waist circumference (cm)	91.3 ± 8.96	74-107	94.0 ± 9.68	78-117	−1.046	0.300
Hip circumference (cm)	101.5 ± 5.06	92-109	103.5 ± 6.02	95-119	−1.271	0.209
Mid-upper arm circumference (cm)	29.5 ± 3.04	22-35	30.0 ± 2.70	24-35	−0.676	0.502
Neck circumference (cm)	35.9 ± 3.54	25-43	37.5 ± 3.71	26-43	−1.567	0.123
Waist/hip ratio	0.9 ± 0.05	0.8-1.0	0.9 ± 0.06	0.8-1.0	−0.780	0.439
**Body composition**						
Body fat ratio (%)	20.1 ± 5.60	10.2-29.8	23.4 ± 6.46	8.5-36.5	−1.918	0.061
Lean body mass (kg)	57.8 ± 5.50	49.8-68.5	58.7 ± 7.29	48.3-73.9	−0.515	0.609
Total body water (%)	56.0 ± 4.63	49.6-64.4	53.6 ± 3.72	48-61.8	2.110	**0.040**

p < 0.05; p values are from the independent-samples t-test.

Table 3 summarizes the daily intake of dietary energy, macronutrients, and micronutrients according to work schedule. Rotating shift workers had a significantly higher total energy intake than fixed daytime workers (2427.8 ± 373.6 kcal vs. 2163.0 ± 340.0 kcal, p = 0.010). Daily nutrient intakes are presented as absolute amounts, with carbohydrate, protein, and fat intakes additionally expressed as percentages of total energy intake and protein intake also expressed as g/kg body weight/day.

**Table 3:** Daily dietary energy, macronutrient, and micronutrient intakes by work schedule.

Nutrients	Fixed Daytime Workers (n = 26)	Rotating Shift Workers (n = 26)	p
Energy (kcal)	2163.0 ± 339.98	2427.8 ± 373.64	**0.010** ^**a**^
CHO (g)	207.1 ± 52.22	232.5 ± 44.95	0.065^a^
CHO (% energy)	39.7 ± 9.67	39.4 ± 6.33	0.893^a^
Sucrose (g)	32.2 ± 22.88	42.6 ± 24.0	0.116^a^
Fructose (g)	9.7 ± 6.34	14.8 ± 6.51	**0.007** ^**a**^
Protein (g)	82.0 ± 15.70	90.0 ± 19.03	0.107^a^
Protein (% energy)	15.6 ± 2.27	15.1 ± 2.10	0.413^a^
Protein (g/kg/day)	1.07 ± 0.21	1.10 ± 0.25	0.545^a^
Fat (g)	98.75 (82.36–107.88)	121.55 (104.60–144.32)	**0.001** ^**b**^
Fat (% energy)	41.7 ± 7.28	45.5 ± 5.93	**0.046** ^**a**^
SFA (g)	37.4 ± 10.43	49.4 ± 13.11	**<0.001** ^**a**^
MUFA (g)	36.6 ± 8.08	43.0 ± 11.19	**0.021** ^**a**^
PUFA (g)	20.1 ± 7.05	21.6 ± 7.01	0.430^a^
Cholesterol (mg)	266.10 (175.43–571.48)	576.60 (464.94–712.05)	**0.001** ^**b**^
Fiber (g)	19.1 ± 6.06	20.8 ± 5.85	0.322^a^
Vitamin A (µg)	861.32 (641.85–1072.59)	1228.58 (981.64–1577.73)	**0.001** ^**b**^
Vitamin E (mg)	16.8 ± 8.80	18.6 ± 6.32	0.420^a^
Thiamine (mg)	0.9 ± 0.19	1.0 ± 0.27	0.055^a^
Niacin (mg)	32.7 ± 7.39	33.3 ± 8.93	0.798^a^
Vitamin B6 (mg)	1.27 (1.13–1.47)	1.41 (1.06–1.67)	0.492^b^
Vitamin B12 (µg)	5.83 (4.10–7.46)	6.82 (4.99–7.41)	0.173^b^
Folate (µg)	300.08 (231.26–363.30)	368.80 (301.59–466.24)	**0.010** ^**b**^
Vitamin C (mg)	82.06 (59.10–127.80)	95.18 (65.47–148.12)	0.431^b^
Iron (mg)	10.9 ± 1.90	13.0 ± 3.37	**0.008** ^**a**^
Magnesium (mg)	322.4 ± 49.20	351.5 ± 82.30	0.127^a^
Zinc (mg)	12.8 ± 3.06	14.3 ± 3.37	0.094^a^
Calcium (mg)	886.3 ± 232.14	1051.3 ± 281.04	**0.025** ^**a**^
Selenium (µg)	5.89 (2.96–34.83)	23.17 (18.46–34.82)	0.067^b^
Phosphorus (mg)	1214.3 ± 177.16	1438.0 ± 352.14	**0.006** ^**a**^
Potassium (mg)	2711.2 ± 375.40	2869.2 ± 697.94	0.314^a^

Data are presented as mean ± SD or median (Q1–Q3), as appropriate.

p < 0.05; ^a^Independent-samples t-test; ^b^Mann–Whitney U test

Among macronutrients, rotating shift workers had significantly higher daily intakes of total fat (p = 0.001), saturated fatty acids (p < 0.001), monounsaturated fatty acids (p = 0.021), and fructose (p = 0.007) than fixed daytime workers. The percentage of total energy derived from fat was also significantly higher among rotating shift workers (p = 0.046). In contrast, carbohydrate and protein intakes did not differ significantly between groups, whether expressed as absolute daily amounts or as percentages of total energy intake; protein intake expressed per kilogram of body weight was also similar between groups (all p > 0.05). Cholesterol intake was significantly higher among rotating shift workers (p = 0.001). Among micronutrients, rotating shift workers had significantly higher daily intakes of vitamin A (p = 0.001), folate (p = 0.010), iron (p = 0.008), calcium (p = 0.025), and phosphorus (p = 0.006). No significant differences were observed for the remaining micronutrients.

Table 4 shows that Dietary Inflammatory Index scores were similar between fixed daytime workers (0.2 ± 1.86) and rotating (–0.2 ± 2.25) shift workers, with no significant difference (p = 0.540). The distribution of participants across DII quartiles (Q1–Q4) was likewise comparable between groups, indicating that the inflammatory potential of the diet did not differ by shift schedule.

**Table 4:** DII scores and DII quartiles according to work schedule.

	Fixed Daytime Workers (n = 26)		Rotating Shift Workers (n = 26)		
Variables	Mean±SD	Min-Max	Mean±SD	Min-Max	p
**DII**	0.2 ± 1.86	−4.6–3.3	−0.2 ± 2.25	−5.3–3.9	0.540^a^
**DII Quartiles**	**n (%)**		**n (%)**		
Q1	4 (30.8)		9 (69.2)		0.429^b^
Q2	8 (61.5)		5 (38.5)		
Q3	7 (53.8)		6 (46.2)		
Q4	7 (53.8)		6 (46.2)		

p < 0.05; p values are from ^a^Independent sample t-test; ^b^Pearson’s chi-square test

Regarding sleep quality, the global PSQI score ranged from 1 to 17, with a mean score of 5.3 ± 3.17 in the overall study population. The mean global PSQI score was 4.7 ± 2.42 among fixed daytime workers and 6.0 ± 3.70 among rotating shift workers. Poor sleep quality (global PSQI score > 5) was observed in 30.8% of fixed daytime workers and 38.5% of rotating shift workers. Neither the mean global PSQI score nor the prevalence of poor sleep quality differed significantly between the groups (both p > 0.05).

Table 5 presents the correlations between DII scores and PSQI component and total scores according to work schedule. Among rotating shift workers, significant positive correlations were found between DII scores and the PSQI sleep duration component score (r = 0.450, p = 0.021) as well as daytime dysfunction component score (r = 0.390, p = 0.049). In the overall sample, significant positive correlations were found between DII scores and the PSQI sleep duration component score (r = 0.319, p = 0.021), daytime dysfunction component score (r = 0.287, p = 0.039), and global PSQI score (r = 0.278, p = 0.046), indicating that DII scores were positively correlated with poorer overall sleep quality, poorer sleep duration component scores, and greater daytime dysfunction. However, no significant correlations were found between DII scores and subjective sleep quality, sleep latency, habitual sleep efficiency, or sleep disturbance component scores (all p > 0.05). In the multiple linear regression analysis, the association between DII score and global PSQI score was not statistically significant after adjustment for age, BMI, smoking status, physical activity, and work schedule (B = 0.369, 95% CI: −0.089 to 0.828; β = 0.240, p = 0.112). Detailed results are presented in Supplementary Table S1 in S1 File.

**Table 5:** Relationship between DII scores and PSQI subcomponents and global PSQI score.

	Dietary Inflammatory Index					
	Fixed Daytime Workers (n = 26)		Rotating Shift Workers (n = 26)		All Workers (n = 52)	
PSQI subcomponents	r	p	r	p	r	p
Subjective sleep quality	0.168^a^	0.411	0.342^a^	0.087	0.234^a^	0.096
Sleep latency	−0.311^a^	0.122	0.249^a^	0.220	−0.010^a^	0.946
Sleep duration	0.159^a^	0.438	0.450^a^	**0.021**	0.319^a^	**0.021**
Habitual sleep efficiency	−0.148^b^	0.470	−0.093^b^	0.650	−0.122^b^	0.389
Sleep disturbance	0.180^a^	0.379	0.251^a^	0.217	0.212^a^	0.131
Daytime dysfunction	0.139^a^	0.498	0.390^a^	**0.049**	0.287^a^	**0.039**
Global PSQI score	0.071^b^	0.730	0.389^b^	0.050	0.278^b^	**0.046**

p < 0.05; p values are from ^a^Pearson correlation test, ^b^Spearman correlation test

Biochemical parameters and blood pressure measures were evaluated for their associations with DII scores. No significant correlations were observed between DII scores and these parameters in either fixed daytime or rotating shift workers (all p > 0.05; Supplementary Table S2 in S1 File).

## Discussion

This study compared dietary inflammatory potential, sleep quality, dietary intake, anthropometric and body composition measurements, and biochemical parameters among male defense industry workers with fixed daytime and rotating shift work schedules. To our knowledge, this is the first study to evaluate these parameters concurrently among defense industry workers in Türkiye.

In the present study, no significant differences in most anthropometric measurements or body composition parameters were observed between fixed daytime and rotating shift workers (p > 0.05). Nevertheless, the mean BMI of rotating shift workers was classified as overweight, whereas that of fixed daytime workers was classified as normal weight. Body fat percentage was also numerically higher among rotating shift workers. Although these differences were not statistically significant, the difference in BMI classification may be clinically relevant, particularly given previous evidence linking shift work with higher BMI and an increased risk of overweight and obesity [41–43].

Consistent with our findings, several observational studies have reported null or weak associations between shift work and anthropometric outcomes, particularly after adjustment for potential lifestyle and occupational confounding factors [44,45]. In contrast, large-scale cohort studies and meta-analyses have reported a higher risk of obesity and less favorable metabolic profiles among shift workers, especially among men with prolonged exposure to rotating schedules and those employed in industrial settings [42,46,47]. In a large cohort of 52,234 workers, prolonged exposure to rotating night work was associated with an increased risk of overall and central obesity among men [48]. Taken together, this heterogeneous body of evidence suggests that the association between shift work and body composition may be context-dependent, and may vary according to shift characteristics, occupational environment, sex, lifestyle behaviors, and methodological differences across studies.

Numerous studies indicate that shift work is associated with unhealthy dietary behaviors, including higher energy intake, greater consumption of carbohydrates and saturated fat, increased intake of energy-dense snacks and sugar-sweetened beverages, and lower fruit and vegetable intake, particularly during night and rotating shifts [18,49–55]. Night shift workers have further been reported to consume chocolate and sugary drinks more frequently as a means of maintaining alertness [50–54]. Consistent with this literature, rotating shift workers in the present study exhibited higher total energy intake and absolute daily intakes of fructose, total fat, saturated fatty acids, monounsaturated fatty acids, and cholesterol.

Similarly, an observational follow-up study demonstrated that energy and fat consumption peaked during 24-hour shift days and declined markedly on subsequent post-shift days [56]. These findings may be related to differences in meal timing and dietary choices across work schedules. Recent evidence suggests that shift work consistently disrupts eating behaviors and chrononutrition patterns, including delayed meal timing, prolonged eating windows, and a greater tendency to consume energy-dense foods, although findings regarding total daily energy intake remain inconsistent across studies [57,58]. Such alterations may contribute to circadian misalignment and adverse metabolic outcomes, thereby increasing long-term metabolic risk among rotating shift workers. However, the dietary differences observed in the present study should be interpreted cautiously given the multiple nutrient comparisons performed.

Beyond macronutrient intake, emerging evidence indicates that shift schedules may also be associated with differences in micronutrient consumption. Studies conducted in night-shift settings in Türkiye have reported alterations in overall diet quality across night work periods, which may indirectly affect micronutrient density through reduced consumption of fruits, vegetables, and other nutrient-rich foods [56]. Population-based evidence has also shown that male shift workers may be at greater risk of inadequate micronutrient intake, including inadequate intakes of thiamine, riboflavin, niacin, calcium, phosphorus, and potassium [59]. Similarly, Nakamura et al. [60] reported higher total energy intake but lower intakes of several micronutrients, including folate, calcium, and iron, among shift workers compared with daytime workers. In contrast, rotating shift workers in the present study had higher absolute daily intakes of several micronutrients. However, given the higher total energy intake in this group, these differences may partly reflect greater overall food consumption and should not necessarily be interpreted as indicating a more favorable micronutrient intake pattern.

Contrary to expectations, no significant differences in Dietary Inflammatory Index (DII) scores or quartile distributions were observed between fixed daytime workers and rotating shift workers. The lack of a between-group difference in DII may partly reflect the standardised institutional meal provision shared by both groups, which could have reduced variability in dietary inflammatory potential. In contrast, analyses of NHANES data have reported higher DII scores among shift workers, particularly among those engaged in rotating schedules [19], whereas no significant differences have been observed in certain occupational groups, such as nursing populations [61]. Despite the absence of a between-group difference in DII in the present study, a recent prospective analysis of hypertensive adults demonstrated that the combination of shift work and a pro-inflammatory diet was associated with a significantly increased risk of all-cause mortality, while adherence to an anti-inflammatory dietary pattern appeared to mitigate these adverse associations [62]. Taken together, these findings suggest that the association between shift work and the dietary inflammatory potential may vary across occupational settings and populations.

The literature indicates that shift work and dietary inflammatory potential may be associated with adverse inflammatory and health-related outcomes [63,64]. Large cohort studies have consistently reported elevated concentrations of CRP, leukocytes, and other inflammatory markers among shift workers, reflecting an increased inflammatory burden [65,66]. Similarly, higher DII scores have been associated with higher triglyceride and LDL cholesterol concentrations and lower HDL cholesterol concentrations [67]. Recent evidence, including observational studies and meta-analyses, further supports associations of higher DII scores with less favorable metabolic and inflammatory profiles, including elevated CRP concentrations and an increased risk of metabolic syndrome and related metabolic abnormalities [67–70]. In contrast, no significant associations were observed between DII scores and inflammatory or metabolic biomarkers in the present study. This finding may partly reflect methodological and contextual factors, including the relatively small sample size, and the homogeneous occupational and dietary environment of the study population, which may have reduced the ability to detect significant associations.

Given the established links between dietary inflammatory potential and systemic inflammation, pro-inflammatory diets may plausibly contribute to disturbances in sleep regulation. In the present study, higher DII scores were associated with poorer overall sleep quality, as reflected by higher global PSQI scores in the overall study population. However, this association was no longer statistically significant after adjustment for potential confounding factors. When work schedules were examined separately, higher DII scores were associated with higher PSQI sleep duration component scores, indicating poorer sleep duration, as well as greater daytime dysfunction among rotating shift workers. No corresponding associations were observed among fixed daytime workers.

Although evidence specifically focusing on shift-working populations remains limited, these findings are partially consistent with previous studies reporting associations between higher DII scores and poorer sleep quality, reduced sleep efficiency, and altered sleep duration [24,25,71,72]. Potential mechanisms underlying these associations may involve systemic inflammation and inflammatory signaling, including IL-6, TNF-α, and CRP [73]. Oxidative stress may represent an additional pathway involved in the relationship between inflammatory processes and sleep disturbances [28]. Furthermore, alterations in melatonin signaling and circadian rhythm disruption may contribute to disturbances in sleep–wake regulation [74]. These mechanisms may be particularly relevant to rotating shift workers, who commonly experience fragmented and irregular sleep patterns in the context of repeated circadian misalignment.

However, not all studies have reported consistent associations between dietary inflammatory potential and sleep outcomes. In particular, some studies have found no significant relationships between DII and sleep quality [75,76]. Such discrepancies may reflect differences in age distribution, work schedules, occupational stress, obesity prevalence, and comorbid health conditions across study populations, underscoring the need for further research in larger and more diverse cohorts.

### Strengths and limitations

The strengths of this study include the simultaneous assessment of the inflammatory potential of the diet (DII), sleep quality (PSQI), anthropometric measurements, and biochemical parameters. This multidimensional approach enabled a comprehensive evaluation of the relationships between shift work, nutrition, and sleep. In addition, the use of validated and reliable measurement tools, together with standardised data collection procedures, enhanced the methodological robustness of the study. Nevertheless, several limitations should be acknowledged. While the sample size was sufficient for the primary analyses, it may have been insufficient to detect smaller effect sizes or support complex multivariable models. Furthermore, the reliance on self-reported measures of dietary intake and sleep quality may have introduced recall and reporting bias.

In addition, residual confounding cannot be excluded, as work-related stress and other unmeasured lifestyle or occupational factors may have been associated with both dietary patterns and sleep quality. The cross-sectional design precludes inference regarding the temporal direction or causality of the observed associations. Finally, the study included only male participants recruited from a single defense industry setting, limiting the generalizability of the findings to women and to workers in other occupational settings. Future studies involving larger and more diverse populations, including both sexes and different occupational groups, and employing longitudinal or prospective designs are needed to further investigate the relationship between dietary inflammatory potential and sleep quality.

## Conclusion

In this study conducted among male employees in the defense industry, rotating shift workers exhibited higher daily energy and fat intakes than fixed daytime workers. However, no significant differences were observed between work schedules in anthropometric measurements, body composition, sleep quality, or DII scores. Across the study population, DII scores were significantly associated with poorer overall sleep quality, as reflected by higher global PSQI scores, higher sleep duration component scores, and greater daytime dysfunction. However, the association between DII and overall sleep quality was no longer statistically significant after adjustment for potential confounding factors. These findings suggest that pro-inflammatory dietary patterns and poor sleep quality may represent co-existing factors associated with metabolic risk, and that addressing both may contribute to better overall metabolic health.

Future research should employ longitudinal or interventional study designs and include larger and more heterogeneous populations to more clearly elucidate potential causal relationships. Additionally, the use of objective sleep assessment methods would strengthen the evaluation of sleep quality and provide more comprehensive evidence regarding the relationship between dietary inflammatory potential and sleep.

## Supporting information

S1 File Supplementary Tables S1 and S2. Supplementary Table S1 presents the multivariable linear regression analysis for total PSQI score. Supplementary Table S2 presents correlations between DII scores and biochemical parameters according to work schedule.
(DOCX)

## References

[1] Deği̇rmenci̇oğlu M. Vardiyalı Çalışma Sistemi Çalışanlar İçin Ne Kadar İyi?. Journal of Aviation. 2019;3(2):113–21. doi: 10.30518/jav.591364

[2] Shan Z, Li Y, Zong G, Guo Y, Li J, Manson JE, et al. Rotating night shift work and adherence to unhealthy lifestyle in predicting risk of type 2 diabetes: results from two large US cohorts of female nurses. BMJ. 2018;363:k4641. doi: 10.1136/bmj.k4641 30464025 PMC6247172

[3] Härmä M, Karhula K, Puttonen S, Ropponen A, Koskinen A, Ojajärvi A, et al. Shift work with and without night work as a risk factor for fatigue and changes in sleep length: A cohort study with linkage to records on daily working hours. J Sleep Res. 2019;28(3):e12658. doi: 10.1111/jsr.12658 29383788

[4] Wang D, Ruan W, Chen Z, Peng Y, Li W. Shift work and risk of cardiovascular disease morbidity and mortality: A dose-response meta-analysis of cohort studies. Eur J Prev Cardiol. 2018;25(12):1293–302. doi: 10.1177/2047487318783892 29929393

[5] Çalık KY, Aktaş S, Bulut HK, Anahar EÖ. The effect of shift and on-call working systems on nurses. J Health Sci Prof. 2015;2(1):33–45.

[6] Srour B, Plancoulaine S, Andreeva VA, Fassier P, Julia C, Galan P, et al. Circadian nutritional behaviours and cancer risk: New insights from the NutriNet-santé prospective cohort study: Disclaimers. Int J Cancer. 2018;143(10):2369–79. doi: 10.1002/ijc.31584 29744870

[7] Hemmer A, Mareschal J, Dibner C, Pralong JA, Dorribo V, Perrig S, et al. The effects of shift work on cardio-metabolic diseases and eating patterns. Nutrients. 2021;13(11):4178. doi: 10.3390/nu13114178 34836433 PMC8617838

[8] Centofanti S, Banks S, Colella A, Dingle C, Devine L, Galindo H, et al. Coping with shift work-related circadian disruption: A mixed-methods case study on napping and caffeine use in Australian nurses and midwives. Chronobiol Int. 2018;35(6):853–64. doi: 10.1080/07420528.2018.1466798 29764218

[9] Kabisch S, Hajir J, Sukhobaevskaia V, Weickert MO, Pfeiffer AFH. Impact of dietary fiber on inflammation in humans. Int J Mol Sci. 2025;26(5):2000. doi: 10.3390/ijms26052000 40076626 PMC11900212

[10] Jiang R, Wang T, Han K, Peng P, Zhang G, Wang H, et al. Impact of anti-inflammatory diets on cardiovascular disease risk factors: a systematic review and meta-analysis. Front Nutr. 2025;12:1549831. doi: 10.3389/fnut.2025.1549831 40181944 PMC11965126

[11] Hepsomali P, Groeger JA. Examining the role of systemic chronic inflammation in diet and sleep relationship. J Psychopharmacol. 2022;36(9):1077–86. doi: 10.1177/02698811221112932 35861213 PMC9516605

[12] Whalen KA, McCullough ML, Flanders WD, Hartman TJ, Judd S, Bostick RM. Paleolithic and mediterranean diet pattern scores are inversely associated with biomarkers of inflammation and oxidative balance in adults. J Nutr. 2016;146(6):1217–26. doi: 10.3945/jn.115.224048 27099230 PMC4877627

[13] Wu P-Y, Chen K-M, Tsai W-C. The Mediterranean Dietary Pattern and Inflammation in Older Adults: A Systematic Review and Meta-analysis. Adv Nutr. 2021;12(2):363–73. doi: 10.1093/advances/nmaa116 33002104 PMC8009747

[14] Zhang Y, Xun L, Qiao R, Jin S, Zhang B, Luo M, et al. Advances in research on the role of high carbohydrate diet in the process of inflammatory bowel disease (IBD). Front Immunol. 2024;15:1478374. doi: 10.3389/fimmu.2024.1478374 39588368 PMC11586370

[15] Tristan Asensi M, Napoletano A, Sofi F, Dinu M. Low-grade inflammation and ultra-processed foods consumption: A review. Nutrients. 2023;15(6):1546. doi: 10.3390/nu15061546 36986276 PMC10058108

[16] Cavicchia PP, Steck SE, Hurley TG, Hussey JR, Ma Y, Ockene IS, et al. A new dietary inflammatory index predicts interval changes in serum high-sensitivity C-reactive protein. J Nutr. 2009;139(12):2365–72. doi: 10.3945/jn.109.114025 19864399 PMC2777480

[17] Shivappa N, Steck SE, Hurley TG, Hussey JR, Hébert JR. Designing and developing a literature-derived, population-based dietary inflammatory index. Public Health Nutr. 2014;17(8):1689–96. doi: 10.1017/S1368980013002115 23941862 PMC3925198

[18] Clark AB, Coates AM, Davidson ZE, Bonham MP. Dietary Patterns under the Influence of Rotational Shift Work Schedules: A Systematic Review and Meta-Analysis. Adv Nutr. 2023;14(2):295–316. doi: 10.1016/j.advnut.2023.01.006 36805319 PMC10229381

[19] Wirth MD, Burch J, Shivappa N, Steck SE, Hurley TG, Vena JE, et al. Dietary inflammatory index scores differ by shift work status: NHANES 2005 to 2010. J Occup Environ Med. 2014;56(2):145–8. doi: 10.1097/JOM.0000000000000088 24451608 PMC3922825

[20] Wang Y, Wang P-C, Wang X-N, Yi L, Diao D-L, Yan B. Shift work and risk of sleep disturbances in occupational populations: a systematic review and meta-analysis. BMC Public Health. 2026;26(1):2254. doi: 10.1186/s12889-026-27636-2 42092856 PMC13430882

[21] Boivin DB, Boudreau P, Kosmadopoulos A. Disturbance of the circadian system in shift work and its health impact. J Biol Rhythms. 2022;37(1):3–28. doi: 10.1177/07487304211064218 34969316 PMC8832572

[22] Coxon C, Nishihira J, Hepsomali P. Dietary inflammatory index, sleep duration, and sleep quality: A systematic review. Nutrients. 2024;16(6):890. doi: 10.3390/nu16060890 38542801 PMC10974932

[23] Buysse DJ, Reynolds CF 3rd, Monk TH, Berman SR, Kupfer DJ. The Pittsburgh Sleep Quality Index: a new instrument for psychiatric practice and research. Psychiatry Res. 1989;28(2):193–213. doi: 10.1016/0165-1781(89)90047-4 2748771

[24] Farrell ET, Hébert JR, Heflin K, Davis JE, Turner-McGrievy GM, Wirth MD. Dietary inflammatory index (DII) and sleep quality, duration, and timing: A systematic review. Sleep Med Rev. 2024;77:101964. doi: 10.1016/j.smrv.2024.101964 38833836 PMC11381171

[25] Golmohammadi M, Samadi M, Salimi Y, Nachvak SM, Ebrahimzadeh Attari V. The association of dietary inflammatory index with sleep outcomes: A systematic review. Health Promot Perspect. 2024;14(2):136–47. doi: 10.34172/hpp.42595 39291049 PMC11403336

[26] Bazmi S, Pourmontaseri H, Shahraki SFM, Pourmontaseri AR, Askari A, Bagheri P, et al. Association between energy-adjusted dietary inflammatory index and sleep quality disorders: a cross-sectional study on fasa adult cohort. J Health Popul Nutr. 2025;44(1):239. doi: 10.1186/s41043-025-00998-w 40618166 PMC12228316

[27] Cho JH, Bhutani S, Kim CH, Irwin MR. Anti-inflammatory effects of melatonin: A systematic review and meta-analysis of clinical trials. Brain Behav Immun. 2021;93:245–53. doi: 10.1016/j.bbi.2021.01.034 33581247 PMC7979486

[28] Davinelli S, Medoro A, Savino R, Scapagnini G. Sleep and Oxidative Stress: Current Perspectives on the Role of NRF2. Cell Mol Neurobiol. 2024;44(1):52. doi: 10.1007/s10571-024-01487-0 38916679 PMC11199221

[29] Wirth MD, Fekedulegn D, Andrew ME, McLain AC, Burch JB, Davis JE, et al. Longitudinal and cross-sectional associations between the dietary inflammatory index and objectively and subjectively measured sleep among police officers. J Sleep Res. 2022;31(4):e13543. doi: 10.1111/jsr.13543 34967055 PMC9240102

[30] Cohen J. Statistical power analysis for the behavioral sciences. 2nd ed. Hillsdale (NJ): Lawrence Erlbaum Associates. 1988.

[31] Kang H. Sample size determination and power analysis using the G*Power software. J Educ Eval Health Prof. 2021;18:17. doi: 10.3352/jeehp.2021.18.17 34325496 PMC8441096

[32] Kushner RF, Gudivaka R, Schoeller DA. Clinical characteristics influencing bioelectrical impedance analysis measurements. Am J Clin Nutr. 1996;64(3 Suppl):423S-427S. doi: 10.1093/ajcn/64.3.423S 8780358

[33] Physical status: the use and interpretation of anthropometry. Report of a WHO Expert Committee. World Health Organ Tech Rep Ser. 1995;854:1–452. 8594834

[34] World Health Organization. Waist circumference and waist-hip ratio: Report of a WHO expert consultation, Geneva, 8–11 December 2008. Geneva: World Health Organization. 2011.

[35] Ben-Noun L, Laor A. Relationship of neck circumference to cardiovascular risk factors. Obes Res. 2003;11(2):226–31. doi: 10.1038/oby.2003.35 12582218

[36] Thompson FE, Subar AF. Dietary assessment methodology. In: Coulston AM, Boushey CJ, Ferruzzi MG, Delahanty LM, editors. Nutrition in the prevention and treatment of disease. 4th ed. San Diego (CA): Academic Press. 2017. p. 5–48.

[37] Rakıcıoğlu N, Tek Acar N, Ayaz A, Pekcan G. Yemek ve besin fotoğraf kataloğu: ölçü ve miktarlar. 3rd ed. Ankara: Hacettepe University Faculty of Health Sciences, Department of Nutrition and Dietetics. 2012.

[38] Shivappa N, Steck SE, Hurley TG, Hussey JR, Ma Y, Ockene IS, et al. A population-based dietary inflammatory index predicts levels of C-reactive protein in the Seasonal Variation of Blood Cholesterol Study (SEASONS). Public Health Nutr. 2014;17(8):1825–33. doi: 10.1017/S1368980013002565 24107546 PMC3983179

[39] Ağargün MY, Kara H, Anlar Ö. Pittsburgh uyku kalitesi indeksinin geçerliği ve güvenirliği. Turk Psikiyatri Derg. 1996;7:107–15.

[40] Liu XC, Tang MQ. Reliability and validity of the Pittsburgh Sleep Quality Index. Chin J Psychiatry. 1996;29:103–7.

[41] Sun M, Feng W, Wang F, Li P, Li Z, Li M, et al. Meta-analysis on shift work and risks of specific obesity types. Obes Rev. 2018;19(1):28–40. doi: 10.1111/obr.12621 28975706

[42] Łagowska K, Kuleta-Koberska A, Michalak M, Bajerska J. The effect of shift work on body mass index: A systematic review and meta-analysis of observational studies. Am J Hum Biol. 2024;36(6):e24041. doi: 10.1002/ajhb.24041 38189567

[43] Liu Q, Shi J, Duan P, Liu B, Li T, Wang C, et al. Is shift work associated with a higher risk of overweight or obesity? A systematic review of observational studies with meta-analysis. Int J Epidemiol. 2018;47(6):1956–71. doi: 10.1093/ije/dyy079 29850840

[44] Harris MA, Kim J, Demers P. Metabolic health measurements of shift workers in a national cross-sectional study: Results from the Canadian Health Measures Survey. Am J Ind Med. 2021;64(11):895–904. doi: 10.1002/ajim.23283 34346078

[45] Dicom AR, Huang X, Hilal S. Association between Shift Work Schedules and Cardiovascular Events in a Multi-Ethnic Cohort. Int J Environ Res Public Health. 2023;20(3):2047. doi: 10.3390/ijerph20032047 36767411 PMC9916120

[46] Soltanzadeh A, Eyvazlou M, Mohammad-Ghasemi M, Sadeghi-Yarandi M, Rahimkhani M, Ghasemi N, et al. Investigating the relationship between shift work schedule and blood and metabolic parameters: a 10-years retrospective cohort study. Sci Rep. 2024;14(1):17297. doi: 10.1038/s41598-024-68378-8 39068300 PMC11283525

[47] Sweeney E, Yu ZM, Dummer TJB, Cui Y, DeClercq V, Forbes C, et al. The relationship between anthropometric measures and cardiometabolic health in shift work: Findings from the Atlantic PATH Cohort Study. Int Arch Occup Environ Health. 2020;93(1):67–76. doi: 10.1007/s00420-019-01459-8 31346709

[48] Bourgkard E, Boini S, Grzebyk M, Dziurla M, Ferrières J, Goldberg M, et al. Duration of exposure to night work and cardiovascular risk factors: Results from 52,234 workers of the CONSTANCES study. BMC Public Health. 2025;25(1):356. doi: 10.1186/s12889-025-21511-2 39875896 PMC11773728

[49] Souza RV, Sarmento RA, de Almeida JC, Canuto R. The effect of shift work on eating habits: a systematic review. Scand J Work Environ Health. 2019;45(1):7–21. doi: 10.5271/sjweh.3759 30088659

[50] Gupta CC, Coates AM, Dorrian J, Banks S. The factors influencing the eating behaviour of shiftworkers: what, when, where and why. Ind Health. 2019;57(4):419–53. doi: 10.2486/indhealth.2018-0147 30404995 PMC6685801

[51] Lin T-T, Guo YL, Gordon CJ, Chen Y-C, Wu H-C, Cayanan E, et al. Snacking among shiftwork nurses related to non-optimal dietary intake. J Adv Nurs. 2022;78(11):3629–40. doi: 10.1111/jan.15253 35429043 PMC9790560

[52] Hemiö K, Lindström J, Peltonen M, Härmä M, Viitasalo K, Puttonen S. The association of work stress and night work with nutrient intake - a prospective cohort study. Scand J Work Environ Health. 2020;46(5):533–41. doi: 10.5271/sjweh.3899 32391566 PMC7737803

[53] Chen C, ValizadehAslani T, Rosen GL, Anderson LM, Jungquist CR. Healthcare Shift Workers’ Temporal Habits for Eating, Sleeping, and Light Exposure: A Multi-Instrument Pilot Study. J Circadian Rhythms. 2020;18:6. doi: 10.5334/jcr.199 33133210 PMC7583716

[54] Lin T-T, Park CG, Kapella MC, Martyn-Nemeth P, Tussing-Humphreys L, Rospenda KM, et al. Shift work domains and their interactions with empty calorie food/beverage consumption: A 14-day intensive longitudinal study. Int J Nurs Stud. 2023;141:104490. doi: 10.1016/j.ijnurstu.2023.104490 37004340 PMC10323862

[55] Navruz-Varlı S, Mortaş H. Shift Work, Shifted Diets: An Observational Follow-Up Study on Diet Quality and Sustainability among Healthcare Workers on Night Shifts. Nutrients. 2024;16(15):2404. doi: 10.3390/nu16152404 39125285 PMC11313754

[56] Navruz Varlı S, Mortaş H. The Effect of 24 h Shift Work on the Nutritional Status of Healthcare Workers: An Observational Follow-Up Study from Türkiye. Nutrients. 2024;16(13):2088. doi: 10.3390/nu16132088 38999836 PMC11243474

[57] Chatterjee R, Sahu S. Navigating Nutrition in a 24/7 World: A Scoping Review of Dietary Intake and Chrononutrition Assessment in Shift Working Populations. J Hum Nutr Diet. 2026;39(3):e70272. doi: 10.1111/jhn.70272 42135909

[58] Saggi MK, Phillips CL, Comas M, Hoyos CM, Marshall NS, Shiao JS-C, et al. Does daily energy and macronutrient intake differ between work and non-workdays in shift workers? A mixed methods study. Chronobiol Int. 2023;40(8):1039–48. doi: 10.1080/07420528.2023.2246559 37599382

[59] Lee W, Jung J, Ahn J, Kim H-R. Rate of inappropriate energy and micronutrient intake among the Korean working population. Public Health Nutr. 2020;23(18):3356–67. doi: 10.1017/S1368980019004075 32183914 PMC10200625

[60] Nakamura M, Miura A, Nagahata T, Toki A, Shibata Y, Okada E, et al. Dietary intake and dinner timing among shift workers in Japan. J Occup Health. 2018;60(6):467–74. doi: 10.1539/joh.2018-0070-OA 30175718 PMC6281632

[61] Molzof HE, Wirth MD, Burch JB, Shivappa N, Hebert JR, Johnson RL, et al. The impact of meal timing on cardiometabolic syndrome indicators in shift workers. Chronobiol Int. 2017;34(3):337–48. doi: 10.1080/07420528.2016.1259242 28107043 PMC5527274

[62] Li Y, Peng X, Wang X, Zhang Y, Liu J, Chen H, et al. Association of shift work and dietary inflammatory potential with all-cause death among US hypertensive population: National health and nutrition examination study, 2005–2010. BMC Public Health. 2023;23:1094. doi: 10.1186/s12889-023-15740-6 PMC10245501 37280597

[63] Phillips CM, Shivappa N, Hébert JR, Perry IJ. Dietary Inflammatory Index and Biomarkers of Lipoprotein Metabolism, Inflammation and Glucose Homeostasis in Adults. Nutrients. 2018;10(8):1033. doi: 10.3390/nu10081033 30096775 PMC6115860

[64] Wirth MD, Shivappa N, Burch JB, Hurley TG, Hébert JR. The dietary inflammatory index, shift work, and depression: Results from NHANES. Health Psychol. 2017;36(8):760–9. doi: 10.1037/hea0000514 28557499 PMC5646681

[65] Matre D, Christensen JO, Mork PJ, Ferreira P, Sand T, Nilsen KB. Shift work, inflammation and musculoskeletal pain-The HUNT Study. Occup Med (Lond). 2021;71(9):422–7. doi: 10.1093/occmed/kqab133 34551112

[66] Woo S-J, Chae C-H, Lim J-W. Association between shift work and inflammatory markers in workers at an electronics manufacturing company. Ann Occup Environ Med. 2022;34:e35. doi: 10.35371/aoem.2022.34.e35 36544883 PMC9748154

[67] Alptekin İM, Ekinci Gezmiş T, Özgeriş FB, Şahin Z, Öztürk N, Orbak Z. Dietary Inflammatory Index and cardiometabolic and inflammatory profiles in children and adolescents with type 1 diabetes: a cross-sectional study. Pediatr Res. 2026;:10.1038/s41390-026-05069–2. doi: 10.1038/s41390-026-05069-2 42104134 PMC13672054

[68] Christodoulou A, Christophi CA, Sotos-Prieto M, Moffatt S, Zhao L, Kales SN, et al. The dietary inflammatory index and cardiometabolic parameters in US firefighters. Front Nutr. 2024;11:1382306. doi: 10.3389/fnut.2024.1382306 38938668 PMC11208711

[69] Hua R, Liang G, Yang F. Meta-analysis of the association between dietary inflammation index and C-reactive protein level. Medicine (Baltimore). 2024;103(19):e38196. doi: 10.1097/MD.0000000000038196 38728463 PMC11081557

[70] Bakhshimoghaddam F, Chaharlang R, Mansoori A, Dehghanseresht N. Dietary inflammatory index and its association with risk of metabolic syndrome and its components: a systematic review and Meta-analysis of Observational studies. J Health Popul Nutr. 2024;43(1):87. doi: 10.1186/s41043-024-00580-w 38898498 PMC11188268

[71] El-Ali Z, Hebert JR, Wirth MD, Mitri R. Association between the Dietary Inflammatory Index and Sleep Quality among Lebanese University Students. Sleep Sci. 2024;17(3):e235–43. doi: 10.1055/s-0044-1780501 39268335 PMC11390170

[72] Godos J, Ferri R, Caraci F, Cosentino FII, Castellano S, Shivappa N, et al. Dietary Inflammatory Index and Sleep Quality in Southern Italian Adults. Nutrients. 2019;11(6):1324. doi: 10.3390/nu11061324 31200445 PMC6627935

[73] Irwin MR. Sleep and inflammation: partners in sickness and in health. Nat Rev Immunol. 2019;19(11):702–15. doi: 10.1038/s41577-019-0190-z 31289370

[74] Baser KHC, Haskologlu IC, Erdag E. Molecular Links Between Circadian Rhythm Disruption, Melatonin, and Neurodegenerative Diseases: An Updated Review. Molecules. 2025;30(9):1888. doi: 10.3390/molecules30091888 40363695 PMC12073486

[75] Masaad AA, Yusuf AM, Shakir AZ, Khan MS, Khaleel S, Cheikh Ismail L, et al. Sleep quality and Dietary Inflammatory Index among university students: a cross-sectional study. Sleep Breath. 2021;25(4):2221–9. doi: 10.1007/s11325-020-02169-z 32829458

[76] Zare MJ, Masoumi SJ, Zare M. The association between energy-adjusted dietary inflammatory index and physical activity with sleep quality: a cross-sectional study. BMC Nutr. 2024;10(1):26. doi: 10.1186/s40795-024-00834-0 38310318 PMC10838418

