# Supplementary material for: Association among Dietary Inflammatory Index, sleep quality and nutritional status in fixed daytime and rotating shift workers
Source: PLoS One. 2026 Oct 22;21(10):e0360075. doi: 10.1371/journal.pone.0360075 (PMC13690223; doi:10.1371/journal.pone.0360075)
Supplement: Supplementary file 1 [file pone.0360075.s001.docx]

**Supplementary Table S1. Multivariable linear regression analysis for total PSQI score**

| **Variable** | **B** | **95% CI** | **SE** | **β** | **p** |
| --- | --- | --- | --- | --- | --- |
| DII score | 0.369 | −0.089, 0.828 | 0.228 | 0.240 | 0.112 |
| Age (years) | 0.060 | −0.074, 0.194 | 0.067 | 0.131 | 0.373 |
| BMI (kg/m^2^) | −0.097 | −0.349, 0.155 | 0.125 | −0.115 | 0.444 |
| Smoking status | −0.528 | −2.003, 0.947 | 0.732 | −0.105 | 0.475 |
| Physical activity | −0.510 | −1.844, 0.825 | 0.663 | −0.116 | 0.455 |
| Work schedule | 1.528 | −0.417, 3.473 | 0.966 | 0.244 | 0.121 |

R² = 0.153; adjusted R² = 0.040; F(6,45) = 1.352; p = 0.255.

Dependent variable: Total PSQI score. B: unstandardized regression coefficient; SE: standard error; β: standardized regression coefficient; CI: confidence interval

**Supplementary Table S2.** **Correlations between DII scores and biochemical parameters according to work schedule**

|  | **Dietary Inflammatory Index** | | | | | | | |
| --- | --- | --- | --- | --- | --- | --- | --- | --- |
|  | **Fixed Daytime Workers (n=26)** | | | **Rotating Shift Workers (n=26)** | | | **All Workers(n=52)** | |
| **Biochemical Parameters** | **r** | **p** | **r** | | **p** | **r** | | **p** |
| Fasting blood glucose (mg/dL) | 0.214^a^ | 0.294 | -0.084 ^b^ | | 0.682 | 0.115 ^a^ | | 0.417 |
| Total cholesterol (mg/dL) | -0.011^b^ | 0.956 | -0.326 ^a^ | | 0.104 | -0.127 ^a^ | | 0.369 |
| HDL-C (mg/dL) | 0.017 ^b^ | 0.934 | -0.113 ^a^ | | 0.581 | -0.058 ^a^ | | 0.682 |
| LDL-C (mg/dL) | -0.009 ^b^ | 0.964 | -0.302 ^a^ | | 0.134 | -0.172 ^a^ | | 0.222 |
| Triglyceride (mg/dL) | 0.002 ^b^ | 0.992 | -0.060 ^b^ | | 0.770 | 0.019 ^b^ | | 0.895 |
| CRP (mg/L) | -0.130 ^a^ | 0.528 | 0.065 ^b^ | | 0.752 | -0.093 ^b^ | | 0.514 |
| Homocysteine (µmol/L) | 0.067 ^b^ | 0.744 | 0.125 ^a^ | | 0.543 | 0.076 ^a^ | | 0.591 |
| SBP (mmHg) | 0.350 ^b^ | 0.242 | -0.193 ^b^ | | 0.491 | -0.091 ^b^ | | 0.645 |
| DBP (mmHg) | -0.142 ^b^ | 0.715 | -0.215 ^b^ | | 0.480 | -0.165 ^a^ | | 0.462 |

p < 0.05 was considered statistically significant. Pearson Correlation Test^a^, Spearman Correlation Test^b^
